# Racial Differences in Dietary Relations to Cognitive Decline and Alzheimer’s Disease Risk: Do We Know Enough?

**DOI:** 10.3389/fnhum.2020.00359

**Published:** 2020-09-03

**Authors:** Puja Agarwal, Martha C. Morris, Lisa L. Barnes

**Affiliations:** ^1^Department of Internal Medicine, Rush Institute for Healthy Aging, Rush University Medical Center, Chicago, IL, United States; ^2^Rush Alzheimer’s Disease Center, Rush University Medical Center, Chicago, IL, United States; ^3^Department of Neurological Sciences, Rush University Medical Center, Chicago, IL, United States

**Keywords:** diet, nutrition, cognition, health disparities, race

## Abstract

The elderly population in the US is increasing and projected to be 44% minority by 2060. African Americans and Hispanics are at increased risk of cognitive impairment and Alzheimer’s disease compared to non-Hispanic whites. These conditions are associated with many other adverse health outcomes, lower quality of life, and substantial economic burden. In the past few decades, diet has been identified as an important modifiable risk factor for cognitive decline and Alzheimer’s disease. Some studies report poor diet quality among African American and Hispanic older adult populations compared to their white counterparts. We have a limited understanding of how diet affects brain health in different racial-ethnic groups. One primary reason for our lack of knowledge is that most cohort studies are of majority non-Hispanic white participants. Moreover, those that do include minority participants do not publish their findings stratified by racial-ethnic groups, and likely have a less accurate measurement of dietary intake among minority groups. In this review, we summarize the current, albeit limited, literature on racial/ethnic differences in dietary relations to dementia outcomes. We will also discuss methodological issues in conducting nutrition studies in diverse cultures, and suggestions for future research directions. Overcoming the gaps will make it possible to make dietary recommendations for Alzheimer’s prevention that are more relevant for different racial/ethnic groups and set us on a faster track to reduce health disparities.

## Introduction

Currently, around 5.7 million Americans have Alzheimer’s dementia, and with a growing aging population, the number is projected to increase to 13.8 million by 2050 (Hebert et al., 2013; Alzheimer’s Association, 2018). The US aging population is also expected to become more racially and ethnically diverse in the coming years, so that by the year 2060, it will be approximately 44% minority (Colby and Ortman, 2014). One systematic review of multi-ethnic cohort studies concluded that dementia incidence rates are higher in African Americans and Hispanics compared to non-Hispanic Whites (Mehta and Yeo, 2017). Some have argued that these observed disparities are due to a multitude of related factors, including increased prevalence of cardiovascular conditions, lower education and socioeconomic status, barriers to health care, and certain lifestyle factors (Judd et al., 2013; Diaz-Venegas et al., 2016; Howard et al., 2018; Weuve et al., 2018). With limited treatments to reverse memory loss or dementia, the identification of modifiable risk factors are of great public health interest. Diet has emerged as a modifiable factor that affects cardiovascular-related conditions but also may have independent effects on the development of dementia. A large body of literature has found that healthy dietary patterns, including the Mediterranean (Scarmeas et al., 2009; Tangney et al., 2011; Koyama et al., 2015; Morris et al., 2015b; Bhushan et al., 2018), DASH (Dietary approach to Reduce Hypertension) (Tangney et al., 2014; Morris et al., 2015b) and MIND (Mediterranean-Dash Intervention for Neurodegenerative Diseases) diets (Morris et al., 2015b,c; Berendsen et al., 2018), and specific foods [e.g., berries (Devore et al., 2012; Agarwal et al., 2019), vegetables (Morris et al., 2006, 2018; Ye et al., 2013a), fish (Morris et al., 2003; Samieri et al., 2018)] and nutrients [vitamin E (Morris et al., 2002a; Beydoun et al., 2015), flavonoids (Devore et al., 2012; Holland et al., 2020), B vitamins (Morris et al., 2005), unsaturated fats (Morris et al., 2004)] are associated with slower cognitive decline and/or reduced risk of dementia. However, the generalizability of these findings to individuals of different race and ethnic backgrounds is not well characterized. We know that diet quality varies by race and ethnicity in the U.S., but differences also occur by socioeconomic status, the region of the country, and urban/rural settings (Aggarwal et al., 2012; Hiza et al., 2013; McInerney et al., 2016; Bloom et al., 2017; Lee-Kwan et al., 2017). Racial/ethnic disparities in dementia may be due, in part, to dietary intakes of the nutrients and foods found to be important to brain health. This was reported to be the case for higher incidence rates of stroke and hypertension among African Americans compared to whites, which were partially explained by higher consumption of a Southern westernized diet pattern among African Americans (Judd et al., 2013; Howard et al., 2018). Unfortunately, in our current state of knowledge, there is limited data to investigate to what extent diet may account for these disparities in dementia. In this review, we will characterize social and biological differences by race and ethnicity that influence diet quality and nutritional metabolism, describe the existing multi-racial/ethnic studies of diet and dementia, and identify the methodological challenges and future directions in closing the gap in this field of scientific inquiry. The existing studies on the association between dietary patterns/food groups/nutrients and cognitive decline/Alzheimer’s dementia risk from the longitudinal cohorts of US adults that included multi-racial/ethnic groups with more than 20% minority population are discussed in this review.

## Racial/Ethnic Differences in Health

Various social and demographic factors that have been studied for health disparities in Alzheimer’s dementia include educational attainment (Weuve et al., 2018), bilingualism (Lamar et al., 2019), neighborhood greenness (Brown et al., 2018), and stressful life events (Zuelsdorff et al., 2020). Similar social and economic aspects may also affect the diet quality by race, including education and income (Raffensperger et al., 2010), health literacy (Kuczmarski et al., 2016), food prices and diet costs (Townsend et al., 2009) and neighborhood grocery store availability (Powell et al., 2007; Bower et al., 2014). Healthy Aging in Neighborhoods of Diversity across the Life Span (HANDLS) study reported lower nutrient-based diet quality among African Americans compared to whites and found health literacy and education as important predictors of diet quality in this urban population (Raffensperger et al., 2010; Kuczmarski et al., 2016). Similarly, African Americans in the Jackson Heart Study reported fast-food clusters (including fast foods, salty snacks, non-diet soft drinks, and meat), as the most common dietary pattern in the study population and was found to be associated with significantly lower levels of plasma carotenoids and alpha tocopherols (Talegawkar et al., 2008). Considering that different social, economic, and demographic factors may influence both diet quality and cognition, examining racial differences in the association of diet with Alzheimer’s dementia and/or cognitive decline, may help us explain, at least in part, health disparities in Alzheimer’s dementia and related disorders.

Although race is socially constructed with little to no basis in biology, there are non-observable differences in nutritional metabolism based on such factors as skin color and body composition (Hall et al., 2010; Bhupathiraju et al., 2011). For example, ultraviolet rays are absorbed by the skin at different rates depending on the level of melanin or pigment in the skin. Racial groups with darker skin pigment require greater sun exposure than lighter-skinned groups to synthesize the same amount of vitamin D (Hall et al., 2010; Gallagher et al., 2013). Differences among racial/ethnic groups in fat and lean body mass can affect the storage and metabolism of a number of vitamins and minerals (Morton et al., 2003; Lear et al., 2009; Travison et al., 2011; Santoro et al., 2018). In the feeding trials, African Americans reported higher, postprandial triglycerides (Goff et al., 2016) and subnormal ghrelin (a gut-brain peptide to signal hunger) suppression (Brownley et al., 2004) compared to Whites. Independent of obesity, body fat distribution, and behavioral factors, African Americans but not Hispanics have high insulin resistance compared to non-Hispanic Whites (Haffner et al., 1996). Genetic variations can also affect susceptibility to DNA damage and DNA repair. For example, African Americans are reported to have lower serum levels of antioxidant nutrients in comparison to whites, yet surprisingly have less oxidative damage to DNA (Huang et al., 2000; Watters et al., 2007). Similarly, a longitudinal analysis indicated higher parathyroid hormones increased diabetes risk only in Whites and not in African Americans (Reis et al., 2016). The growing evidence of differences in nutrition metabolism by race and ethnicity is a compelling reason for their scientific exploration in the dementia field.

## Multi-Racial/Ethnic Cohort Studies on Diet and Dementia

A large body of literature over the past two decades has established diet as an important modifiable risk factor for dementia in older populations. Various healthy dietary patterns [e.g., Mediterranean (Aridi et al., 2017), DASH (Tangney et al., 2014), MIND (Morris et al., 2015c)], foods (Morris et al., 2006, 2016, 2018; Devore et al., 2012; van de Rest et al., 2016; Samieri et al., 2018) and nutrients (Morris et al., 2002a,b, 2003, 2004, 2005, 2018; Haan et al., 2007; Moorthy et al., 2012; Beydoun et al., 2018b; Schneider et al., 2018) have been associated with slower cognitive decline and lower dementia risk in prospective cohort studies. The healthy dietary pattern may exert a neuroprotective effect by reducing oxidative stress and inflammation (Bagetta et al., 2020) and was found to be associated with less brain atrophy (Gu et al., 2015). These dietary patterns are plant-based consisting of foods such as fruits, berries, vegetables, leafy greens, whole grains, fish, olive oil, legumes, and nuts. These foods are rich in essential nutrients as well as bioactive that have anti-inflammatory and antioxidant properties (Ellis et al., 2011; Alvarez-Suarez et al., 2014; Giampieri et al., 2014). *In vitro* and *in vivo* evidence indicate that bioactive and some of their metabolites can cross the blood-brain barrier (Youdim et al., 2003, 2004) and through signal transduction cascades may directly act on neurons and glia (Jaeger et al., 2018). Animal studies reported berries and leafy greens improve cognitive function via increased neurogenesis, and insulin-like growth factor-1 signaling, and reversed neuronal aging by reducing oxidative stress (Joseph et al., 1998; Shukitt-Hale et al., 2015; Elkhadragy et al., 2018). Additionally, these dietary patterns also limit the consumption of red meat, fatty foods, and sweets. Another set of evidence indicate the high fat/cholesterol diet’s deleterious effect on cognition via its effect on synaptic integrity, increased hippocampal insulin resistance, inflammation (Arnold et al., 2014; Denver et al., 2018), as well as increased levels of amyloid precursor protein (Thirumangalakudi et al., 2008).

The association of diet with cognitive decline and Alzheimer’s dementia risk has emerged as important factor that may have huge public health impact on aging population. However, there is a paucity of information on how these associations may differ by race or ethnicity. Two primary reasons emerge to explain this gap. First, not all cohort studies include diet assessment, and those that do are almost exclusively of non-Hispanic white populations. Second, the few multi-racial/ethnic cohort studies that include diet rarely report the findings stratified by racial/ethnic groups (Koyama et al., 2015; Beydoun et al., 2018b), although some report p-values for tests of interaction of the findings by these groups as discussed below.

Table 1 summarizes the findings from nine multi-racial/ethnic cohort studies in the US that report diet associations with dementia outcomes. The studies are large, ranging from 1,956 to 18,080 participants, and the percentages of minority participants provide sufficient sample sizes to observe most diet associations with the outcomes (percentages of minority groups range from 22 to 100%). The non-white groups represented in these studies are African American and Puerto Rican. There is limited (Ye et al., 2013a,b) or non-existent data for Mexican, Native American, and Asian populations. The findings of studies on African Americans and Puerto Ricans cannot be assumed to apply to these other racial/ethnic groups as there are large cultural and social differences, including dietary practices.

**TABLE 1:** Summary of studies on association of Dietary Patterns with cognitive decline or Incident AD that have at least > 20% minority population included.

Cohort and study population	Minority population%	Years Follow-up	N; Mean Age (*SD*)	Exposure	Outcome	Findings	Exposure Interaction with Race
WHICAP- Washington Heights-Inwood Columbia Aging Project (Multi-racial) 68% Female	African American (34%) and Hispanics (34%)	Longitudinal, 4–5 years	*N* = 2258; 77.6 (6.6)	Mediterranean diet	Incident MCI Progression of MCI to AD	**↓** MCI risk and **↓** risk for MCI conversion to AD (Scarmeas et al., 2009)	Not reported
				Healthy dietary pattern for study population *	Incident AD	**↓** AD risk (Gu et al., 2010)	*No stratified analysis by race*
				Total calories and Fat intake	Incident AD	**↑** AD risk (Luchsinger et al., 2002)	
				Antioxidant vitamin	Incident AD	No association (Luchsinger et al., 2003)	
CHAP-Chicago Health and Aging Project	African American (63%)	Longitudinal, 6–9 years	*N* = 3790; 75.4 (6.2)	Mediterranean diet, HEI-2005	Cognitive decline OR Incident AD	Mediterranean diet **↓**cognitive decline (Tangney et al., 2011)	Diet*race not significant *No stratified analysis by race*
				Fruits and Vegetables		Vegetable intake **↓** cognitive decline (Morris et al., 2006)	Diet*race not significant
				Fish intake		n-3 FA **↓** AD risk (Morris et al., 2003)	Diet*race not significant
				Antioxidant vitamins		Vitamin E from foods **↓** cognitive decline (Morris et al., 2002b) **↓** AD risk (Morris et al., 2002a)	Diet*race not significant
				Folate Vitamin B12		**↑** Cognitive decline (Morris et al., 2005) **↓** Cognitive decline (Morris et al., 2005)	Diet*race not significant
				Dietary fats		Saturated fats **↑**cognitive decline (Morris et al., 2004)	Animal fat*race was not significant.
				Serum Vitamin B12		**↓** Cognitive decline (Tangney et al., 2009)	Vitamin B12* race was not significant
				Homocysteine		No association	Homocysteine* race was not significant
				Methyl malonic acid		**↑** Cognitive decline (Tangney et al., 2009)	Methyl malonic acid* race was not significant.
Health ABC – Health Aging and Body Composition Study (Biracial)	African American (38%)	Longitudinal, 8.0 years	*N* = 2326; 74.6 (2.9)	Mediterranean diet	Cognitive decline	**↓** Cognitive decline in Blacks not Whites (Koyama et al., 2015)	Mediterranean diet*race was significant *Stratified analysis by race*
Health and Retirement study 60% women	African American (22%)	Cross-sectional	*N* = 5907; 68 (10.8)	Mediterranean diet	Cognitive Scores	“ + ” Cognition (McEvoy et al., 2017)	Not reported
				MIND diet		“ + ” Cognition	
Coronary Artery Risk Development in Young Adults (CARDIA)	African American (45%)	Longitudinal, 8.0 years	*N* = 2621; 25 (3.5)	Mediterranean diet	Cognitive Scores assessed 25 and 30 years later	↑ Cognitive function in midlife (McEvoy et al., 2019)	Not reported
57% female				DASH diet		No association	
				*A Priori* Dietary Quality Index		↑ Cognitive function in midlife (McEvoy et al., 2019)	
REGARDS- REasons for Geographic And Racial Differences in Stroke	African American (31%)	Longitudinal 4–7 years	*N* = 18,080; 64.4 (9.1)	Plant-based diet Southern diet	Incident cognitive Impairment	↓ Incident cognitive impairment (Pearson et al., 2016) ↑ Incident cognitive impairment (Pearson et al., 2016)	Diet*race not significant *No stratified analysis by race*
				Mediterranean diet		↓ Incident cognitive Impairment in non-diabetic participants (Tsivgoulis et al., 2013)	Only Diet*diabetes significant. Stratified by diabetes status
HANDLS (Healthy Aging in the Neighborhood of Diversity Across Lifespan) Participants (Biracial) 57% female	African American (51%)	Cross-sectional	*N* = 2090; 47.9 (9.2)	HEI-2010	Cognitive Scores	“ + ” Cognition only in those below the poverty line (Beydoun et al., 2018a)	Diet*race not significant *No stratified analysis by race*
				Dietary Antioxidant vitamins		Vitamin E “ + ” Cognition (Beydoun et al., 2015)	Vitamin E*race not significant *No stratified analysis by race*
		Longitudinal, 4–5 years		Dietary Vitamin D	Cognitive decline	↓ Cognitive decline (visual memory) (Beydoun et al., 2018b)	Vitamin D* race interaction significant: Improved visual memory only in Whites and not in Blacks
				Nutrient adequacy score (NAS) Caffeine Alcohol		“ + ” Cognition ↓ Attention decline (Beydoun et al., 2014) “ + ” Cognition “ + ” Attention and Working memory (Beydoun et al., 2014)	NAS*race not significant *No stratified analysis by race*
Two Boston based cohorts (Boston Puerto Rican Health Study (BPRHS) and Nutrition, Aging and Memory in Elders (NAME) study)	African American (37%) BPRHS- Hispanics (100%)	Cross-sectional	*N* = 1956,	Plasma Vitamin B12 Vitamin B6 Folate Homocysteine	Cognitive Scores	“ + ” Cognition (Moorthy et al., 2012) “ + ” Cognition No association No association	Not reported *No stratified analysis by race*
BPRHS (Boston Puerto Rican Health Study), 70% female	Hispanics (100%)	Cross-sectional	N = 1269, 57.3 (7.6)	Mediterranean diet HEI-2005	Cognitive Impairment	“–”cognitive impairment (Ye et al., 2013b)	N/A
				Fruits and Vegetables		“–” Cognitive Impairment (Ye et al., 2013a)	
		Longitudinal 2 years		Dietary n-3 and n-6 PUFA	Cognitive decline	EPA, DHA and n3VLCFA **↑** Executive Function (Bigornia et al., 2018)	
				Plasma vitamin B-6		**↓** Cognitive decline (Palacios et al., 2019a)	
				Serum vitamin D		No association (Palacios et al., 2019b)	

↑ Upward arrow indicates statistically significant increased risk in longitudinal analysis; ↓ downward arrow indicates statistically significant decreased risk in longitudinal analysis; “ + ” Plus indicates statistically significant positive association in the cross-sectional analysis; “−” Minus indicates statistically significant negative association in the cross-sectional analysis. *Dietary pattern for study population identified based on Reduced Rank Regression.

As shown in Table 1, the multi-racial/ethnic cohort studies have a number of positive findings for dementia outcomes and nutrients (vitamin E, vitamin D, folate, vitamin B12, dietary fats), foods (vegetables, fish, caffeine, alcohol), and diet patterns. Some of the studies provide no information about whether the findings were analyzed by race/ethnicity (McEvoy et al., 2019), but in those that do, the findings are not stratified by racial/ethnic group. This is most likely because in nearly every case, tests for interaction effects by race/ethnicity are not statistically significant, although there are a few exceptions. The Health, Aging and Body Composition Study (Health ABC) reported a protective association of the Mediterranean diet with slower cognitive decline in African American but not in whites (Koyama et al., 2015), and a cross-sectional study from the HANDLS (Beydoun et al., 2018b) found a positive association of dietary vitamin D and better visual memory in whites but not in African American. Even though most of these studies did not find statistically significant differences by race or ethnicity, the possibility of heterogeneous effects of diet on brain health by race remains. As discussed below, one must question whether dietary behaviors among the minority participants have been well characterized in these studies.

## Methodological Issues

It is not enough to implement a standardized diet questionnaire into a multi-racial/ethnic cohort study and expect to elicit valid findings on diet and dementia by racial/ethnic groups. The diet assessment tool must represent the foods, cooking methods, recipes and portion sizes that are relevant to the population under study. For diet assessment tools, one size does not fit all. Food frequency questionnaires (FFQ) are the primary method of diet assessment in large epidemiological studies of chronic conditions. They provide a measure of long-term intake that is most relevant to conditions with long latency. This is in contrast to other methods, such as biochemical measures or 24-h dietary recall and diet recording, that may not be good representations of more habitual diet, particularly those nutrients that have high day-to-day variability (Willett, 2013). Although FFQs have been used as a valid and reproducible tool of dietary intake for many years, they are not generalizable beyond the populations for which they have been developed. This is particularly true for racial/ethnic groups as most FFQs were developed for non-Hispanic white populations. FFQs have a predefined list of food items, to which participants respond regarding usual frequency of intake, and for some FFQs, usual portion size (e.g., small, medium, large). The list of foods and their corresponding frequencies and portion sizes vary among FFQs. Two FFQs that have been widely adopted for use by many of the cohort studies are those developed by Willett et al. (1985) (used in the Nurses’ Health Study and Health Professional Follow-up Study) and Block et al. (1986) (used in the National Health and Nutrition Examination Survey, or NHANES). Both the Willett and Block FFQs have been well-validated by biochemical measures and other assessment methods of dietary intake, but their development and validation have been primarily in majority white populations. Thus, implementation of these tools in study populations that include other racial and ethnic groups without validating them raises concerns as to the validity of the study findings. It is possible that the absence of racial/ethnic differences in the diet-dementia findings in Table 1 is due in part to the lower validity of the diet assessment tools to capture intake in the minority groups in some cohorts. Only a few of the cohort studies conducted validation studies of the FFQ within their study populations. Of these few, the validation correlations were somewhat moderate for Chicago Health and Aging Project participants (average *r* = 0.41 in African American vs. *r* = 0.51 in whites for 15 nutrients), and the WHICAP studies (*r* = 0.40 for 7 nutrients, correlations not reported by race/ethnicity). To validly assess diet, the FFQ should capture the most commonly consumed food items for a group as well as culture-specific recipes, cooking methods, and portion sizes. For example, Hispanics consume a bigger rice portion in one meal compared to whites and African American (Tucker et al., 1998). The standard portion size for rice among Puerto Ricans may be 1 cup versus 1/2 cup in non-Hispanic whites (Tucker et al., 1998). Food preparations, preference, cooking methods and recipes of dishes may vary too. For example, one study documented that soul food, a common dietary pattern found in African American culture, contains mainly pork, pork fat, chicken, organ meats, corn, sweet potatoes, and greens (Sucher and Kittler, 2004). The diets of different race/ethnicities in a study population will be well measured only to the extent that the appropriate foods, portions and preparation methods are accurately captured by the assessment method; for example, the Boston Puerto Rican Health Study uses a validated FFQ specifically designed and processed as per the Puerto Rican dietary habits. Similarly, the Jackson Heart Study used a validated FFQ developed based on regional food patterns rather than on the national patterns for whites and African Americans in the lower Mississippi Delta region (Tucker et al., 2007). Thus capturing diet using a validated tool for multi-ethnic populations is an important gap that can be improved in the field of diet and dementia.

The majority of studies on nutrition and dementia outcomes primarily based their findings on dietary intake levels of nutrients and foods. However, our knowledge about nutritional effects on the brain would be greatly enhanced by the addition of biochemical measures. There may be differences in nutrient absorption, metabolism, or delivery to tissues that require different intake levels by race/ethnicity for optimum brain function and disease prevention. The use of biochemical measures in conjunction with dietary intake assessments could be used to better inform public policy on recommended dietary intake levels by race. The reporting of dementia outcomes by level of nutrient intake as well as biochemical level is crucial to advance the field as well as to establish public health and clinical recommendations. Published studies rarely present this information stratified by race or ethnic group; even fewer report biochemical assessments.

Currently, there is much attention on the Mediterranean diet for dementia prevention. However, the promotion of one diet pattern for diverse cultures around the world may not be optimal from the perspective of public health or the environment. It is becoming evident that there are multiple diet patterns favorable to brain health. Given the challenges in achieving behavior change over the long-term, it does not appear feasible to expect individuals to adopt a single diet from a foreign culture that involves introducing new foods that are strange to one’s usual cultural practices, or the elimination of favorite meal items. This approach to behavior change has a high likelihood of failure, particularly if the changes are more expensive. A “one diet” approach to health also would not prove favorable for environmental health. The ecological or carbon footprint may be unnecessarily large due to shipping and storage, particularly if there are local foods and diets that are equally beneficial for maintaining brain health. By studying nutrition and brain health in different cultures, regions, and races, we can identify multiple brain-healthy diets within a region and group. This is another large gap in the field.

## Future Research

In order to advance the field on nutrition and dementia in minority populations, it is imperative that the few studies with large minority populations report estimates of effect stratified by racial/ethnic group, at the very least in supplemental tables. In addition, new multi-racial/ethnic cohort studies are needed that include culturally appropriate and validated diet assessments as well as biochemical measures of nutrient status. Currently, there is limited data on a number of large minority populations in the U.S., particularly those originating from Mexico and other Latin American countries, Asia, and Native Americans. The inclusion of diverse cultures in a study lends to a greater range of nutrient intake levels and thus improved ability to observe diet-dementia relations. The diversity in dietary practices may also lead to discoveries of new nutrients and foods that are important in the disease process. Additionally, we need to understand how cognition is related to the nutrigenomics and nutrigenetics, i.e., two-level interaction between nutrients and genomics. Firstly, nutrients may affect transcriptional factors and modify the gene expression. Secondly, the genetic variability may define the interaction between nutrients and the disease (Fenech et al., 2011; Peña-Romero et al., 2018). Precision nutrition is gaining popularity for other disease outcomes, and future studies in the field of nutrition and cognition focusing on the genetic factors that may alter the relation of various foods with the brain health are needed. The emerging science on the gut microbiome and the gut-brain axis is a new frontier in the dementia field that would be greatly enhanced by diversity in diet that comes with the inclusion of multiple cultures within a study. Another frontier is the measurement of nutrients and metabolites in human brain tissue (Morris et al., 2003, 2015a) and their relations to measures of brain neuropathology. To date, these rare studies have largely been restricted to non-Hispanic whites. Finally, the first diet intervention trials on cognitive health have been initiated. Efforts to test diet approaches in multiple racial/ethnic groups is imperative for better understanding of the disease process and for more effective public health policies to reduce racial/ethnic disparities in dementia.

## Author Contributions

PA: manuscript preparation and critical review. MM and LB: manuscript preparation and critical review of the manuscript for intellectual content. All authors contributed to the article and approved the submitted version.

## Conflict of Interest

The authors declare that the research was conducted in the absence of any commercial or financial relationships that could be construed as a potential conflict of interest.
